# Increased Endothelin-1 Levels of BAL Fluid in Patients with Behçet's Disease

**DOI:** 10.1155/2007/93726

**Published:** 2007-03-19

**Authors:** Kamel Hamzaoui, Hanene Chelbi, Mariam Kamoun, Imen Ben Dhifallah, Agnes Hamzaoui

**Affiliations:** ^1^Homeostasis and Cell Dysfunction Unit Research 99/UR/08-40, Medicine University of Tunis, Tunis 1007, Tunisia; ^2^Pneumology Hospital A. Mami, Department of Paediatric and Respiratory Diseases, Pavillon B, Ariana 2080, Tunisia

## Abstract

*Objective and background*. Pulmonary aneurysms and thrombosis constitute a significant cause of morbidity and mortality in Behçet's disease (BD). Various factors have been studied to explore the pathogenesis of vascular involvement in BD. As endothelin (ET) is known for its potent vasoconstrictor and proinflammatory properties, we supposed that it is involved during the inflammatory process of BD pulmonary vasculitis. *Methods*. To investigate the role of ET in BD, ET-1 concentrations were measured in bronchoalveolar lavage fluid (BALF) of 18 nonsmoking BD patients with pulmonary manifestations and 12 control subjects. Immunoreactivity of ET-1 was also evaluated in alveolar macrophages (AMs) cytoplasm. *Results*. ET-1 levels in BD-BALF were significantly higher than those of controls. ET-1 levels were correlated with the number of alveolar macrophages, but not with BAL-CD4/CD8 ratio. ET-1-immunoreactivity was found mainly in AM of BD-BAL. *Conclusions*. Increased ET-1 production from AM is associated with pulmonary BD manifestations.

## 1. INTRODUCTION

Behçet's disease (BD) is a multisystem inflammatory disorder characterized by recurrent oral ulcers, genital ulcers, and ocular inflammation, and frequently involves the joints, skin, gastrointestinal tract, central nervous system (CNS), and pulmonary manifestations [1–3]. BD is recognized as a multisystem vasculitis, which can affect all types of blood vessels [1]. During the course of BD, 25–37% of patients develop vascular complications [1–4].

Recent evidence suggests that ET should be considered to be an inflammatory mediator [5, 6] and can stimulate macrophages and monocytes to release proinflammatory cytokines. Recent data also indicated that endothelial dysfunction (ED) is a constant feature of BD [7]. Etiology of ED in BD is probably multifactorial. Activation of both innate and Th1-type adaptive immune responses with enhanced expression of proinflammatory cytokines, adhesion molecules, free oxygen radicals [8], and high homocysteine levels [7] have all been suggested responsible for the activation of endothelial cells irrespective of overt vascular manifestations. Chambers et al. [9] observed an increase in FMD (brachial artery flow-mediated endothelium-dependent dilation) values following vitamin C administration in 12 patients, suggesting an important role of oxidative stress in endothelial dysfunction in BD. Inflammatory microenvironment in BD may cause reduced bioavailability of nitric oxide through proinflammatory cytokines as well as increased oxidized LDL cholesterol levels [10, 11].

ET has both mitogenic and proliferative actions on fibroblasts [12] in vitro and is produced by many types of lung cells such as endothelial cells [13], epithelial cells [14] and macrophages [6]. Interestingly, a recent study using human ET-1 transgenic mice has demonstrated that pulmonary ET-1 overexpression causes lung fibrosis [15]. ET-1 also induces fibronectin expression in human bronchial epithelium via an ETA receptor [16], suggesting a contribution of ET-1 as the intermediate pathogenesis of fibrosis because fibronectin is a potent chemotactic factor for fibroblasts [16].

On this background, our study was planned to assess the contribution of bioactive ET to the pulmonary BD manifestations. For this purpose, ET concentration in BALF from BD patients was measured in BAL from active BD patients having pulmonary manifestation.

## 2. MATERIALS AND METHODS

### 2.1. Patients

We studied 18 active BD patients (12 males and 6 females; nonsmoking patients; mean age: 49.8 years; range: 28–52 years). They were selected on the basis of the criteria defined by the International Study Group for diagnosis of Behçet's disease [17]. Clinical features of the patients with active stage are given in Table 1, which describe overall burden of the disease manifestations in BD patients. All active BD patients have pulmonary manifestations. Active BD patients were always treated with steroids and colchicine. The control subjects consisted of 12 nonsmokers (five men and seven women; mean age: 46.2 years; range: 32–48 years) undergoing routine investigations for suspected bronchial carcinoma and whose chest X-ray (CXR), bronchial examination, and pulmonary functions were normal. None of them had evidence of acute infection or chronic disease (e.g., other autoimmune or atopic disorders). Informed consent was obtained from all of the patients and control subjects. The design of the study was approved by our National Ethics Committee.

### 2.2. Bronchoalveolar lavage

Bronchoscopy was performed according to standard guidelines, as described previously [18]. Thirty minutes prior to the procedure patients received 0.5 mg of atropine and 12.5 mg codeine intramuscularly. Local anaesthesia of the oropharynx was achieved by Novesine spray (Wander, Switzerland) until gag reflexes subsided. Bronchoscopy was performed using a Pentax bronchoscope through which 150 ml of normal prewarmed saline in aliquots of 20 ml were instilled into a subsegment of the right middle lobe. BAL fluid was then immediately aspirated by gentle hand suction into plastic tubes and kept at 4°C on ice.

### 2.3. Processing of BAL cells

BAL samples were filtered through a two-layer sterile gauze into sterile plastic vials (Falcon, Oxnard, CA, USA), centrifuged at 4°C and 500 grams for 10 minutes. The supernatant was removed and cells were washed twice in PBS. The total cell number was counted using a Neubauer hemocytometer (Brand, Wertheim, Germany). Differential cell counts were performed after Giemsa staining (Merck, Darmstadt, Germany) of cell smears with 1000 cells per slide counted.

### 2.4. Analysis of T-lymphocyte subsets in BAL

BAL cells were incubated in the presence of saturating concentrations of fluorescein-conjugated MoAb against the surface markers CD3, CD4, and CD8 and isotype-matched control antibodies labelled with FITC, PE, and PE/CY5 (obtained from Dako, Hamburg, Germany) for 20 minutes at room temperature in the dark. Nonspecific fluorescence was detected by incubating mouse IgG of the same isotype, but with irrelevant antigen specificity. After two washes with PBS, the cells were analyzed by flow cytometry (FACScan; Becton Dickinson, Heidelberg, Germany).

### 2.5. Extraction of ET and enzyme immunoassay

Endothelin levels in BALF were assessed as described previously [19]. Briefly, the BALF sample (5 mL, thawed at 4°C) was loaded onto Sep-Pak C18 column (Millipore, Milford, MA, USA) that had been preactivated by methanol, and washed with 0.1% trifluoroacetic acid. Bound ET was eluted with methanol : water : trifluoroacetic acid (90 : 10 : 0.1) and evaporated by centrifugal freeze dryer. The resulting pellet was reconstituted with 500 *μ*L of assay buffer, and then the concentration of extracted ET was measured using Endothelin Enzyme Immunoassay kit (R&D System Europe, Ltd., Abingdon Science Park, UK). The detection limit of the assay was 0.78 pg/mL.

### 2.6. Immunocytochemistry

BAL cells were centrifuged at 300 g for 10 minutes. The slides were air-dried and fixed in acetone. BAL cells were stained with rabbit mAb to human ET-1 (Biotrend Chemicals GmbH; Köln, Germany), using the unlabeled antibody method (indirect method). The slides were incubated for 1 hour at 37°C with a 1/200 dilution of rabbit mAb to human ET-1, and incubated with horseradish peroxidase, labeled goat anti-rabbit IgG for a further 1 hour at 37°C. Peroxidase was visualized by incubating slides in 3,3′-diaminobenzidine tetrahydrochloride and hydrogen peroxide (H_2_O_2_) for 2 minutes.

### 2.7. Measurement of albumin

The concentration of albumin in BALF was measured by ELISA kit (Exocell, Philadelphia, PA, USA). The assay followed a conventional ELISA format, using an antibody that recognizes antigen (albumin) in the test samples.

### 2.8. Statistical analysis

Data are presented as mean ± SE. Comparison of ET-1 levels in BALF between normal subjects and active BD patients was performed using the Student *t* test for paired samples. Correlations were tested by calculation of Pearson's correlation coefficient (*r*). Calculations were performed using the SPSS (10.0). *P* values of <.05 were considered statistically significant.

## 3. RESULTS

### 3.1. BAL differential cell count and CD4/CD8 ratio

BALF recovery volumes were not different between active BD and healthy controls (52.7 ± 9.6% versus 53.4 ± 10.2%; *P* = .38). The BAL cell count tended to be higher in active BD than healthy controls, but did not reach statistical significance (Table 2). The percentage of lymphocytes was higher in active BD-BAL than in control subjects [(28.7 ± 10.2%) versus (16.3 ± 8.5%); *P* = .0002]. The percentage of 12 macrophages was not significantly different between active BD and healthy controls [(68.7 ± 8.4%) versus (72.4 ± 10.7%); *P* = .08]. No differences were observed in the percentages of neutrophils and eosinophils.

The percentage of the CD4^+^ lymphocytes in BAL of patients with active BD (68.7% ± 12.3%) was higher than that of normal controls (52.7% ± 18.5%; *P* = .037). The CD4/CD8 ratio, in patients with active BD (2.26 ± 0.8), was significantly increased when compared to healthy controls (1.16 ± 0.3; *P* = .041).

### 3.2. ET-1 levels in BALF

**Figure 1:**
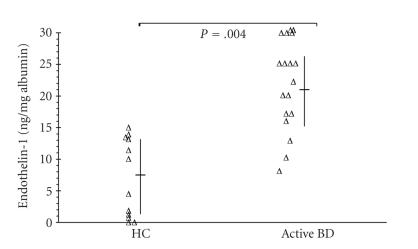
Endothelin-1 concentration in BAL fluid from patients with active Behçet's disease (BD) and from healthy controls (HC). ET-1 levels were measured by enzyme immunoassay and were corrected by an albumin concentration in recovered lavage fluid. Values were expressed as median and the 25th–75th percentile. *P* values are also indicated (Student *t* test) (*P* = .004; *t* = −3.620; ddl: 11).

Endothelin-1 levels in BALF from active BD were significantly higher in active BD than those from healthy controls (22.78 versus 7.23 ng/mg albumin, *P* < .004) (Figure 1).

### 3.3. Correlation between ET-1 concentration and number of alveolar macrophages (AMs) in BALF

**Figure 2:**
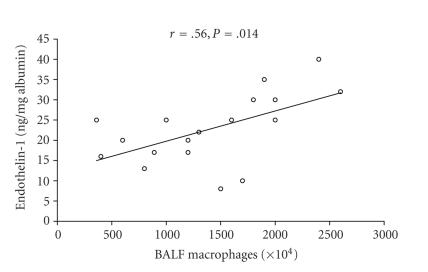
Correlation between endothelin-1 concentrations and the number of alveolar macrophages in BAL fluid from 18 active BD patients with pulmonary manifestations. (*P* = .014; *r* = .56).

**Figure 3:**
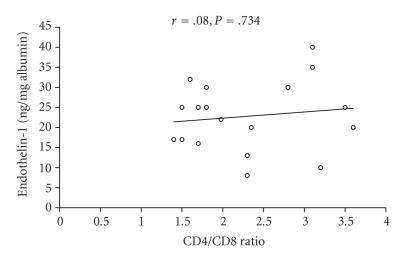
Correlation between endothelin-1 concentrations and CD4/CD8 ratio of alveolar macrophages in BAL fluid from 18 active BD patients with pulmonary manifestations. (*P* = .734; *r* = .08).

There was a positive correlation (*r* = .56; *P* = .014) between the number of macrophages and ET-1 levels in BALF (Figure 2). No correlation (*r* = .08; *P* = .734) was found between ET-1 levels and CD4/CD8 ratio (Figure 3).

### 3.4. Immunocytochemistry of ET-1

**Figure 4:**
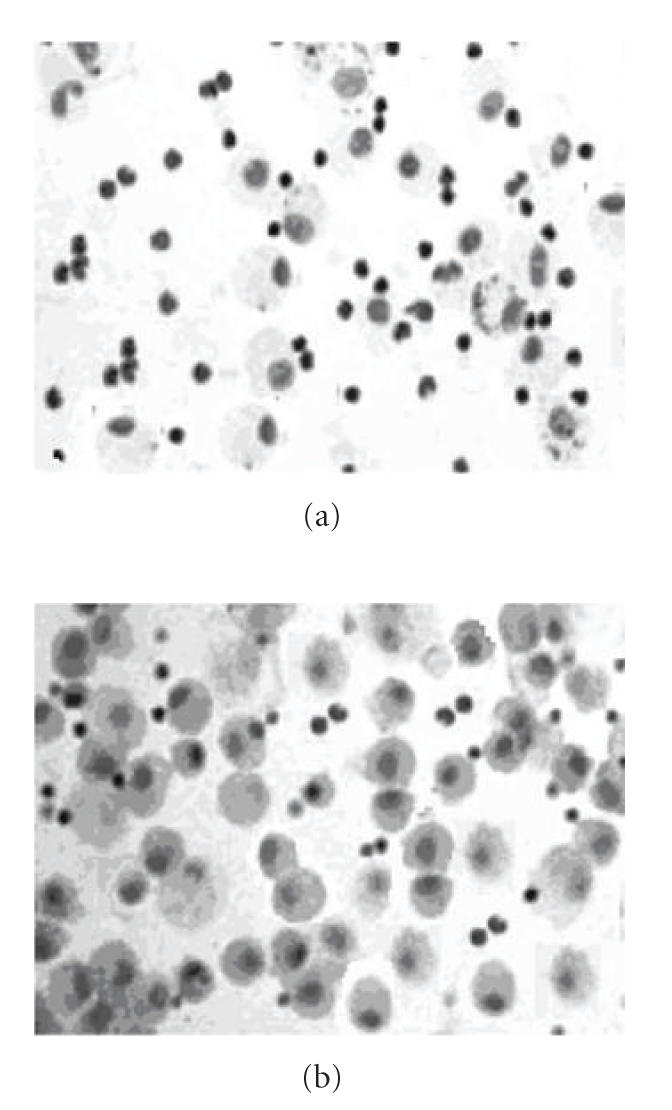
Immunocytochemical detection of endothelin-1 in BAL cells from control subjects (a), and from patients with active BD (b) using rabbit mAb to human endothelin-1. Cytoplasm of BALF cells from patients with active BD was strongly stained. The immunostaining also demonstrated that most of the immunopositive cells in the BD BALF were macrophages.

The cytoplasm of BALF cells from patients with active BD was strongly stained with anti-ET-1, compared to healthy controls. The most stained cells in active BD were macro-phages (Figure 4). The percentage of AMs immunopositive for ET-1 was assessed in five fields and all of the macrophages were immunopositive for ET-1 in active BD BAL (84.77 ± 12.49%), whereas <2% were positively stained in the healthy controls (Table 2). When macrophages were incubated for 24 hours without stimulation, culture media in active BD contained higher levels of ET-1 than for healthy controls.

## 4. DISCUSSION

Pulmonary manifestation in BD is one of the leading causes of death. Mean survival after the onset of hemoptysis was reported to be about 10 months [20, 21]. Recent reports of elevated serum concentrations of von Willebrand factor, plasminogen activator inhibitor-1, and thrombomodulin suggest that impaired vascular endothelial function contributes to vascular pathology in BD [22]. Endothelium-independent vasodilatation (EIVD) was found to be low in patients with BD [7].

The source of ET-1 in the lung has been controversial. In the normal lung, ET-1 is known to be produced by several lung cells such as endothelial cells, epithelial cells, and AM [5]. Increased expression of immunoreactive ET-1 and ET-1 mRNA in pathological conditions including pulmonary manifestations has been demonstrated in AM and proliferating alveolar epithelial cells [5, 23].

In this study, a significant correlation was observed between ET-1 concentrations and the number of AM. An increased presence of immunoreactive ET-1 in cytoplasm of AM from BD patients but not healthy controls was revealed by immunocytochemical assay. These data suggest that ET-1 could play an important role in inflammatory lung manifestations in these patients.

A variety of mediators, which could be involved in the intermediate pathogenesis of lung inflammation in BD patients, has been studied. Proinflammatory cytokines such as TNF-*α*, IFN-*γ*, IL-1, IL-6, IL-8, and IL-18 are known to amplify the inflammatory response in BD [18, 24–26]. Macrophage inhibitory protein-1*α* (MIP-1*α*), a C−C chemokine, which stimulates the activation and migration of leukocytes, was found elevated in patients with BD, and correlates with IL-8, Rantes, and MCP-1 levels [27]. The expression of ET-1 mRNA was increased after incubation with *α*-(IL-8 and MGSA/Gro*α*) and *β*-chemokines (MCP-1, MIP-1*α*, MIP-1*β*, and RANTES), and a mixture of 3 proinflammatory cytokines TNF-*α*, IL-1*β*, and IFN-*γ* was able to increase the mRNA expression of ET-1 [28]. These data provide several findings, the most significant of which is a stimulatory role for chemokines on the vascular endothelium. Our data support a local production of ET-1 within the pulmonary alveoli. In our study, most of the AMs expressed intracytoplasmic ET-1. This finding may be linked with the polarization of T-lymphocytes toward the Th1-type in BD as Th1 cytokines promote acceleration of macrophage function. Furthermore BAL fluid cells from patients with BD show increased mRNA expression of IFN-*γ* and IL-18 compared with normal controls [18, 29]. In the same way, TNF-*α* increases the expression of ET-1 in bronchial epithelial cells, and TNF-*α* release is itself increased by ET-1 [30]. As a matter of fact, high TNF-*α* levels have been observed in BD sera, positively correlated with IL-18 production [31, 32].

The interaction between ET and various cytokines may regulate/exacerbate the pathological events in pulmonary manifestations in BD. ET-1 has been launched as an important mediator in bronchi [33]. In animal studies, the increase in ET-1 mRNA level in lung tissue preceded the increase in mRNA levels of TNF-*α*, IL-1*β*, and IL-8. Treatment of the animals with the ET receptor antagonist bosentan resulted in a substantial decrease in the concentrations of TNF-*α*, IL-1*β*, interferon-*γ*, and ET-1 in BAL fluid [33]. In BD, the interplay between ET-1 and proinflammatory mediators must be elucidated.

Vascular endothelial dysfunction is a central event in the pathogenesis of a variety of human diseases, including adult respiratory distress syndrome, atherosclerosis, and diffuse systemic inflammatory disorders [34]. Endothelial injury seems to induce a dysfunction in nitric oxide (eNOS; iNOS) activity, resulting in a proinflammatory environment in association with tissue damage [34]. Recently, it has also been reported that reduced plasma nitric oxide (NO) levels in BD patients are implicated in the development of the endothelial abnormalities and thrombotic complications occurring in these patients [35]. An impairment of endothelium-dependent, flow-mediated dilatation has been described in BD suggesting a decreased endothelial NO activity, which may contribute to vascular lesions in BD. However, there have been conflicting reports about serum NO concentrations in patients with BD [36]. Recently, endothelial nitric oxide synthase gene Glu298Asp polymorphism was significantly associated with BD in a randomly selected group of Turkish patients [37]. The authors discussed the fact that with respect to the impaired NO production, the significant association of Glu298Asp polymorphism with BD may have a clinical implication as this polymorphism generates protein products with differing susceptibility to cleavage. They discussed the fact that this might have contributed to abnormally low NO generation. The combination of the paracrine effects of ET-1 along with release of NO is likely to have a relevant physiological role in the regulation of blood flow, as ET-1 and NO have opposite effects. ET-1 induces vasoconstriction and thrombosis contributing to ocular pathological manifestations and promoting retinal capillary nonperfusion and *ischaemia* [38]. Increased levels of VEGF and MCP-1 detected in BD thrombosis suggest the possible role of those angiogenic cytokines together with ET-1 in the pathogenesis of the disease. Moreover, ET-1 has mitogenic properties, with animal studies indicating a role in the proliferation of cultured airway smooth muscle cells and airway epithelial cells. The presence of endothelin-1 in increased concentration in BD patients' lungs suggests that further investigation of its role in pulmonary vessel remodeling is justified and potential interactions of endothelin-1 with other growth factors in BD remain to be clarified.

## Figures and Tables

**Table 1:** Clinical features of patients with Behçet's disease (BD) with pulmonary manifestations. Overall burden of the disease manifestations in BD patients is described. Patients received colchicine, steroids/cyclosporine. Five patients with oral ulcer, genital ulcer, uveitis, and vasculo-symptoms were newly diagnosed. All patients had pulmonary aneurysms.

	Number of patients (%)

**Major criteria**
Oral ulcer	18 (100)
Genital ulcer	18 (100)
Skin lesions (erythema nodosum, folliculitis)	14 (77.8)
Uveitis	16 (88.9)
**Minor criteria**
Arthritis	15 (83.3)
Vascular symptoms	09 (50.0)
Intestinal symptoms	05 (27.8)

**Table 2:** BAL fluid results in patients with active Behçet's disease and control subjects. Data are expressed as mean ± SEM.

Alveolar macrophage data	Active BD^(†)^	Controls subjects^(†)^	*P* value^(‡)^

Cells per millilitrex 10^4^	18.6 ± 2.4	13.7 ± 4.7	.092
% Alveolar macrophages	68.7 ± 8.4	72.4 ± 10.7	.08
Albumin (mg/L)	29.6 ± 3.2	18.7 ± 2.3	.07
% ET-1 immunopositive	84.77 ± 12.49	1.92 ± 0.87	.00006

^(†)^Data are from 18 BALs performed in 18 active BD patients and 12 BALs performed in 12 control subjects.

^(‡)^Mann-Whitney *U* test.
